# Design and investigation of charge plasma-based TMD heterojunction TFET biosensor for ultrasensitive detection

**DOI:** 10.1038/s41598-024-84677-6

**Published:** 2025-04-30

**Authors:** Monika Kumari, Niraj Kumar Singh, Vinay Kantipudi, Manodipan Sahoo

**Affiliations:** 1 Department of Electronics Engineering, Government Polytechnic, Asthawan, Nalanda, Bihar 803107 India; 2 Department of Electronics Engineering, Government Polytechnic, Gopalganj, Bihar 841428 India; 3 Department of Electronics Engineering, Indian Institute of Technology (Indian School of Mines), Dhanbad, 826004 India

**Keywords:** Nanoscale devices, Nanoscale materials, Sensors and biosensors

## Abstract

In this work, a charge plasma TMD heterojunction tunnel FET-based dielectrically modulated biosensor is designed and investigated for biosensing applications. In the proposed biosensor, WTe_2_ and MoS_2_ serve as the source and channel material, respectively to form the heterojunction. Whereas the channel-drain junction is a homojunction formed by MoS_2_. The advantage of heterojunction has been exploited to overcome the low I_*ON*_ and ambipolar behavior of TFET, which results in the enhancement of sensitivity. The charge plasma doping has been utilized to mitigate random dopant variations, reduce manufacturing expenses, and simplify the fabrication process. Non-equilibrium green’s function (NEGF)-based simulator and SILVACO TCAD, a 2-D device simulator have been utilized to simulate the electrical characteristics of the proposed biosensor. Uniform filling of the cavities in biosensors is not always practically possible; thus, the issue of partial hybridization is also considered in this work. The proposed biosensor (for k = 9) achieves a high sensitivity of 10^10^, an I_*ON*_/I _*OFF*_ ratio of 10^14^, and a low subthreshold swing of 39 mV/decade. Finally, the proposed biosensor is benchmarked with contemporary works of the literature and it has been observed that the presented charge plasma TMD heterojunction TFET (CP-TMD-HJ-TFET)-based biosensor has emerged to have a superior sensitivity (i.e. I_*ON*_/I_*OFF*_ ratio) which is ∼ 4 decades higher than the maximum sensitivity reported by any contemporary biosensor.

## *Introduction*

FET-based biosensors have had a tremendous impact on various industries, including healthcare, mining, and agriculture, due to their label-free detection, faster response, and high sensitivity. The first FET-based biosensor was an Ion-sensitive FET that was studied by Bergveld et al. in 1970^1^. It can sense the charged biomolecules effectively, however, it can’t detect the neutral biomolecules^2^. Ion-sensitive FETs have several drawbacks, which have led to the development of dielectric modulated FETs. Dielectric modulated FET-based biosensors can detect both charged and neutral biomolecules. The scaling of FET for miniaturization and improvement in the performance of biosensors lead to the following constraints: high response time because of the subthreshold swing (SS) limit, Drain induced barrier lowering (DIBL), short channel effects, low I_*ON*_/I_*OFF*_ ratio, high leakage current etc^3–5^. Because TFETs rely on tunneling rather than thermionic emission, they perform better and provide enhanced performance, resulting in improved sensitivity for a biosensor. Low I_*ON*_ and ambipolar behavior are disadvantages of TFET^6^. The use of a heterojunction with an appropriate band offset helps increase I_*ON*_ and suppress the device’s ambipolar behavior^7,8^. Transition metal dichalcogenides (TMDs), one of the most exciting 2-D semiconducting materials for next-generation biosensors, have recently made significant strides. The use of 2-D semiconductors as a channel material can improve the short channel behavior of TFETs^9^. Greater gate control over the channel is made possible by these 2-D materials, increasing the electric field at the source-channel junction. In addition, 2-D materials have a lower surface roughness than 3-D materials due to their comparably weak interlayer connections^10^. Unlike Graphene, TMD materials have an appreciable band-gap. Thus, TMD-TFETs exhibit an excellent I_*ON*_/I_*OFF*_ ratio which is essential for biosensing applications.

Because of subsequent thin layer, conventional doping is not possible in 2-D semiconductor biosensors. The disadvantages of conventional doping techniques include high manufacturing costs, erratic dopant variations, and complex fabrication^11^. Although electrostatic doping (ED) is possible, this method is not energy-efficient as it requires multiple supply voltages for drain and source doping in these structures^12^. To fix these issues, charge plasma-based TFET devices were suggested. Manufacturing is made simple by the charge plasma method, which dopes the source and drain regions with the proper metals^13^.

In contrast to completely filled cavities, Kim et al.^14^ have described practical occurrences of partially filled cavities caused by steric hindrance. The effective dielectric constant of the cavity decreases in the partially filled cavity scenario because air is present alongside the biomolecules, therefore impacting the efficiency of the biosensor^15^. To the best of the author’s knowledge, the effectiveness of the Charge Plasma TMD heterojunction TFET as a biosensor has not yet been comprehensively studied. As a result, this study provides the first in-depth investigation of the CP-TMD-HJ-TFET-based biosensor for biosensing applications. A breakdown of the paper structure is provided below. The device architecture and simulation methodology are explained in Section II. The simulation results of the suggested biosensor are thoroughly addressed in Section III. Finally, Section IV draws conclusions of the work.

## Device structure

**Fig. 1:**
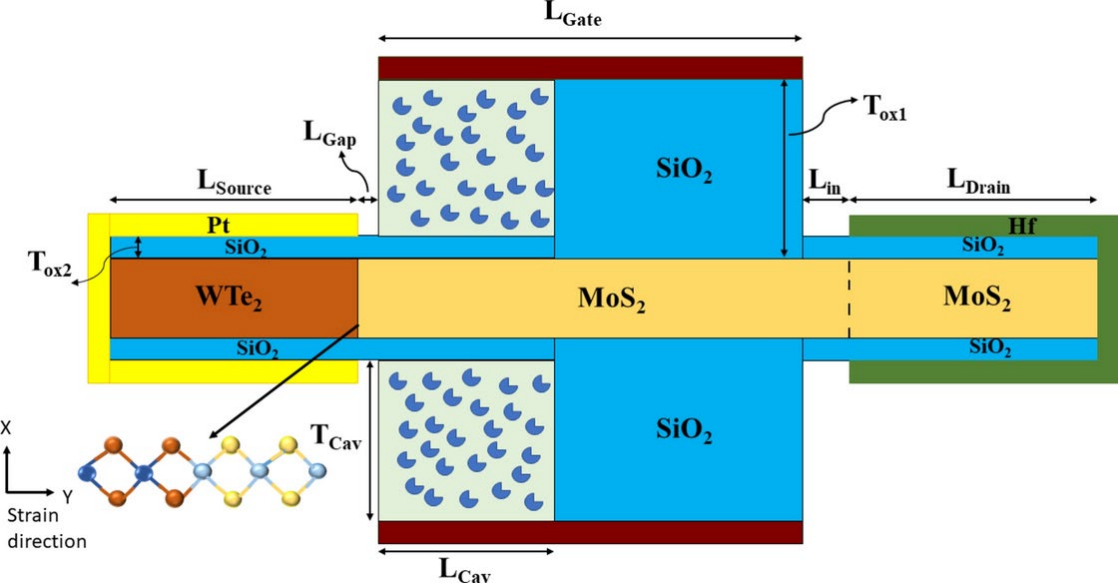
Device architecture of charge plasma TMD heterojunction TFET-based biosensor.

Architecture of the proposed CP-TMD-HJ-TFET-based biosensor is presented in (Fig. 1). A n+ drain, intrinsic channel, and p+ source have been employed in the device. For heterojunction TFET, larger band-gap material in the channel and drain, and material with lower band-gap in the source have to be considered^16^. When the gate to source voltage (V_*gs*_) value is negative, the inclusion of larger band-gap material in the channel and drain regions prevents holes from flowing from drain to channel, thereby suppressing ambipolar behavior. Additionally, having a lower band-gap material in the source side escalates the probability of BTBT of electrons across the source-channel junction, causing higher ON current^8^. Thus, monolayer WTe_2_ is utilized in the source, and monolayer MoS_2_ is employed in the channel and drain region. In the charge plasma doping technique, for n+ doping in the drain region, the work-function of drain metal electrode must be smaller than the work-function of the material in the drain (*ϕ*_*m,d*_ < *χ*_*d*_ + *E*_*g,d*_/2), where, *ϕ*_*m,d*_ is the drain electrode’s work-function, *χ*_*d*_ is the electron affinity of the drain material and *E*_*g,d*_ is the band-gap of drain material^17,18^. The work-function of source metal electrode must be greater than the work-function of the material in the source area to achieve p+ doping in the source (*ϕ*_*m,s*_ > *χ*_*s*_ + *E*_*g,s*_/2), where *ϕ*_*m,s*_ is the source metal electrode’s work-function, *χ*_*s*_ is the electron affinity of the source material and *E*_*g,s*_ is the band-gap of source material^17,18^. Since, Platinum (Pt) and Hafnium (Hf) having work-functions 5.93 eV and 3.9 eV respectively, met the criteria of charge plasma concept, they were used as source and drain metal electrodes respectively. The channel thickness should be smaller than the Debye-length to satisfy the criteria for the charge-plasma concept. Here, the channel thickness is not more than the Debye-length since the monolayer TMDs are considered. The rest of the device parameters are shown in (Table 1).

**Table 1:** Device parameters of the proposed structure.

Symbol	Parameters	Value (nm)
L_Source_	Source-length	40
L_Channel_	Channel-length	60
L_Drain_	Drain-length	40
L_Gate_	Gate-length	53
L_Cav_	Cavity-length	23
T_Cav_	Thickness of the Cavity	9
T_ox1_	Thickness of SiO_2_	10
T_ox2_	Thickness of SiO_2_	1
L_gap_	Gap between the cavity and the Source metal electrode	2
L_in_	Space between drain metal electrode and gate oxide	5

**Fig. 2:**
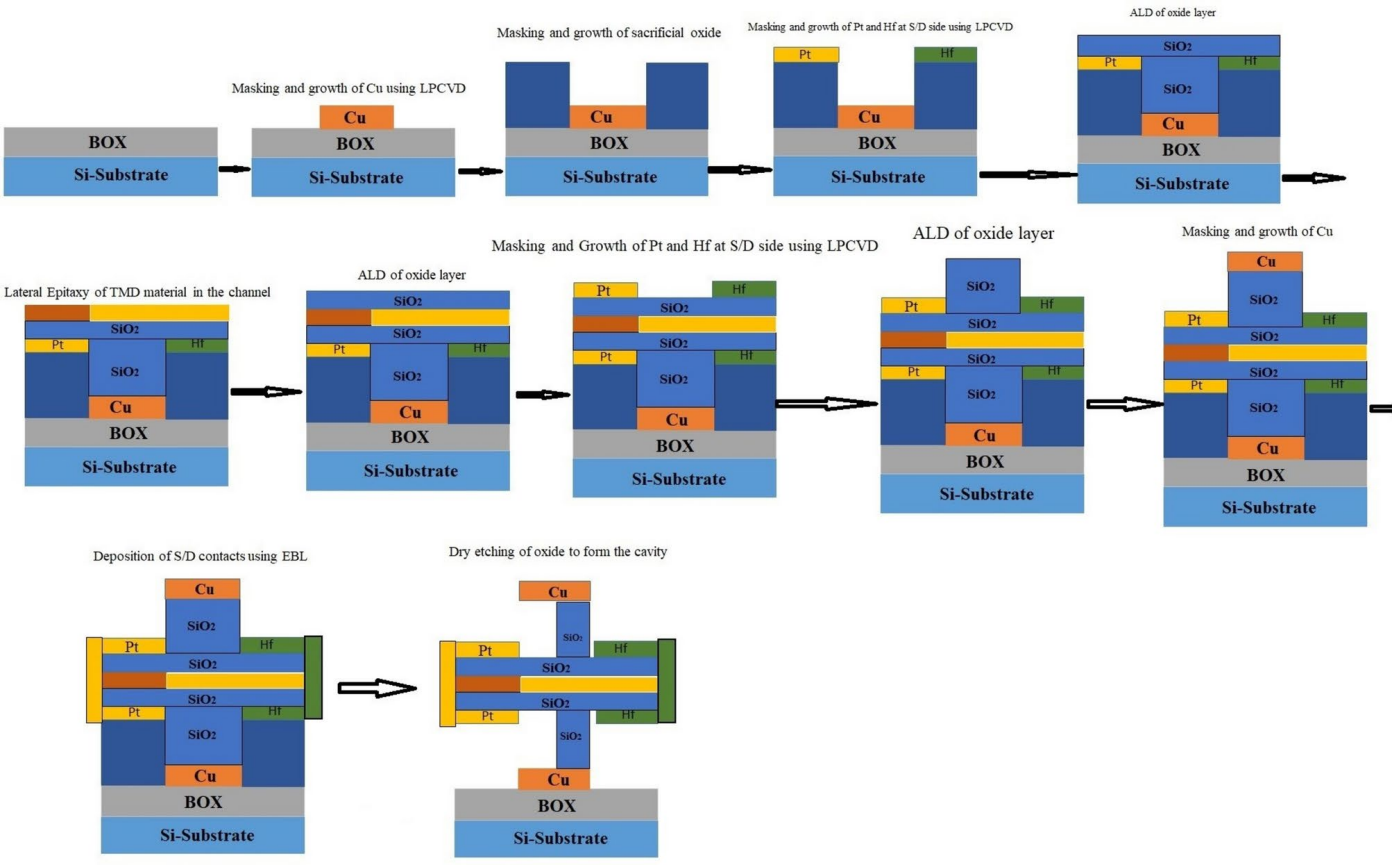
Tentative fabrication steps for charge plasma TMD heterojunction TFET-based biosensor.

At the experimental level, fabrication of a Charge Plasma-based P-N diode has previously been demonstrated^19^. Despite the potential challenges in fabricating CP-TFETs, we can leverage validated processes for similar devices, as documented in references^12,19^, to develop a feasible fabrication plan for realizing CP-TFETs as illustrated in (Fig. 2). It is possible to fabricate the suggested structure using photolithography, EBL and dry etching. At first, patterning is done over the substrate then the gate metal is deposited using LPCVD^11^, then EBL is used to create the sacrificial layer. Using CVD^19^, Pt and Hf metals are deposited over the source and drain region as anode and cathode. After this, ALD is used to deposit the oxide layer^12^. Then, the mechanically exfoliated monolayer layer of WTe_2_ and MoS_2_ can be laterally grown in the channel utilizing edge epitaxy^20^. Electrodes for the source and drain can be grown via LPCVD. Finally, the sacrificial layer is eliminated, and cavities are created carefully by wet-etching the oxide layer. To replicate biomolecules having different dielectric constants (k) viz. Streptavidin (k=2.1), Biotin (k=2.63), Bacteriophage T7 (k=6.3), APTES (k=3.57), Ferro-cytochromec (k=4.7) and Keratin (k=8)^3,11,15^, materials having dielectric constants between k=1 (air) to k=9 are considered for analysis. Furthermore, charge density from *ρ* = 5×10^−11^ to *ρ* = 5×10^11^ C/cm^2^ is considered to demonstrate the effects of charged biomolecules of different concentrations in the cavity.

## Results and discussions

**Fig. 3:**
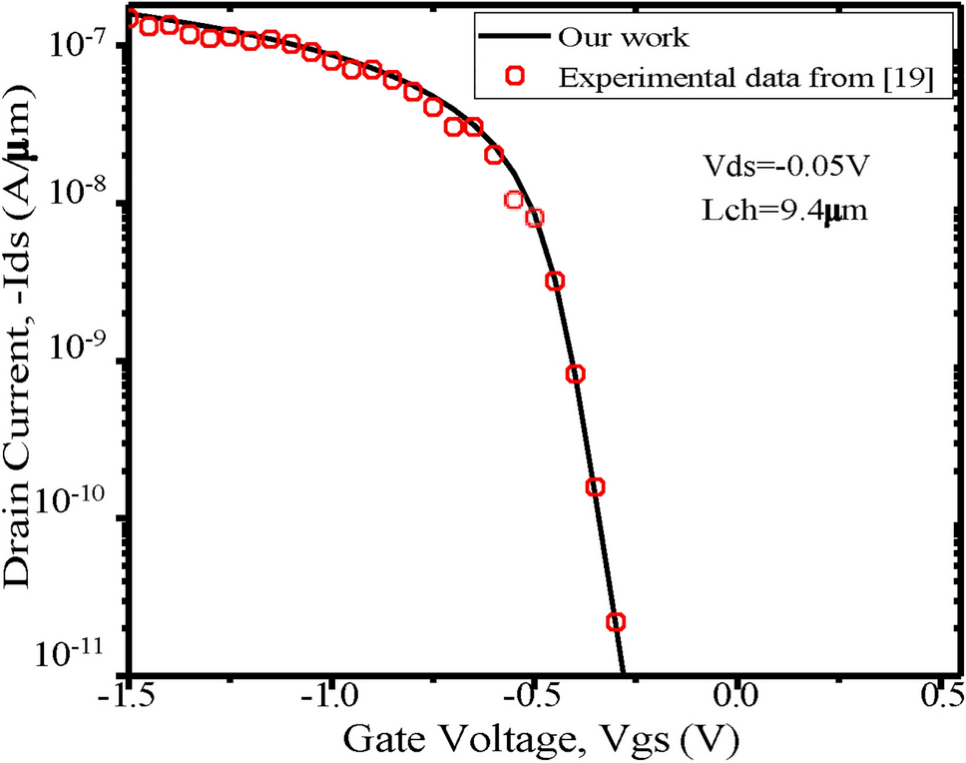
The drain current validation of the simulated TMD FET with the experimental data from^21^.

In this section, the characteristics of the proposed biosensor are discussed in detail. This study is the first to investigate the charge plasma TMD Heterojunction TFET-based biosensor, hence, there is not any literature to support the suggested architecture. Therefore, the simulation framework is cross-validated using existing data from TMD FET and TFET studies. To confirm its validity against experimental data, the architecture is adjusted to resemble a TMD FET, and the simulated results closely match the experimental findings from^21^ as displayed in (Fig. 3). Minor disparities between the simulated and experimental data are attributed to variations in the experimental setup and simulation environment. Additionally, the charge plasma TMD Homojunction TFET-based biosensor is simulated using NanoTCAD ViDES and validated with SILVACO TCAD.

**Fig. 4:**
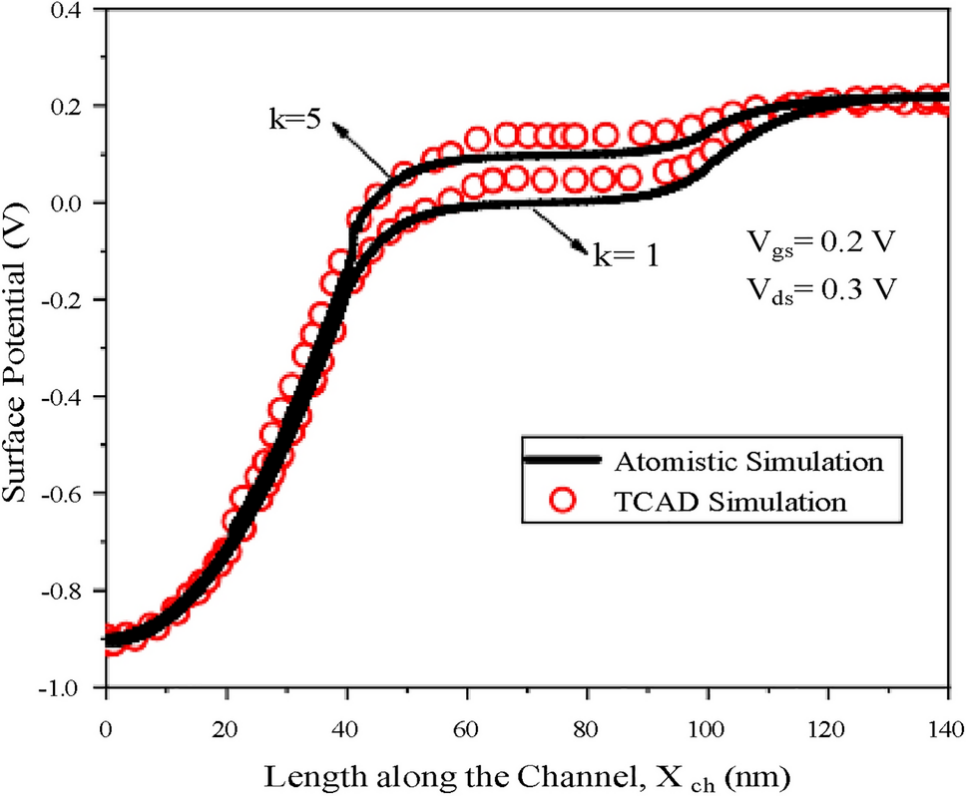
Surface potential plot of charge plasma homojunction TFET-based biosensor for dielectric constant k = 1, 5 at V_*gs*_ = 0.2 V, V_*ds*_ = 0.3 V.

**Fig. 5:**
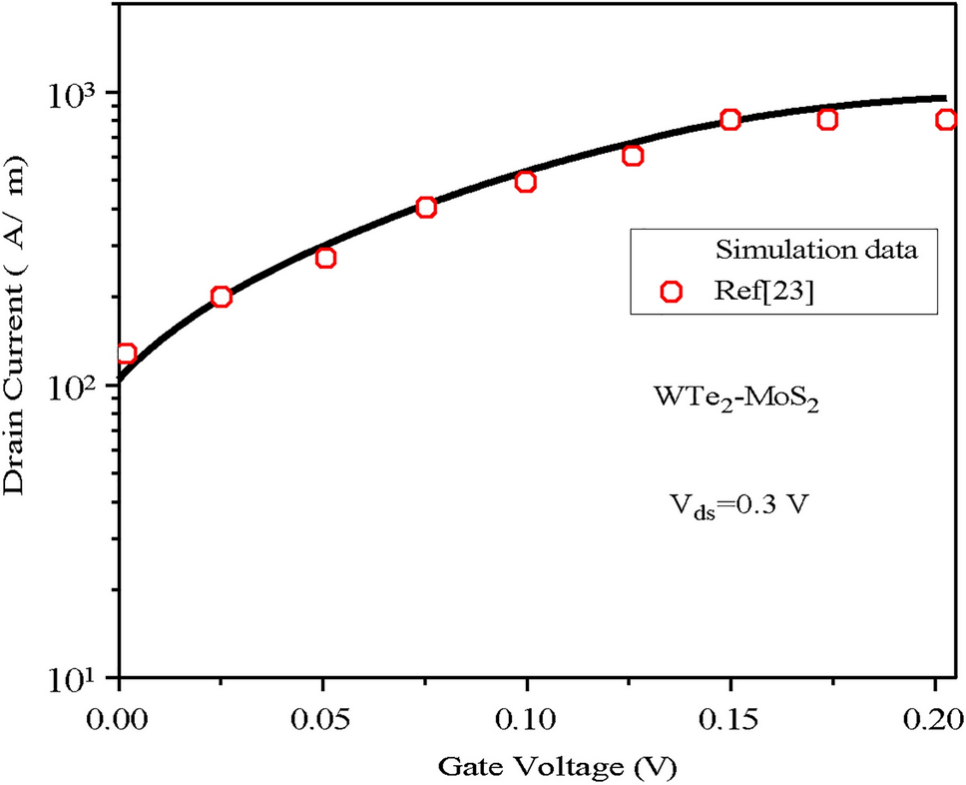
The Drain Current validation of the simulated Heterojunction TMD TFET with the data from^16^.

The charge plasma TMD Homojunction TFET-based biosensor is simulated with NanoTCAD ViDES a NEGF simulator and the surface potential at different dielectric constants is validated with SILVACO TCAD as shown in (Fig. 4). Moreover The simulation data of the proposed heterojunction TMD TFET has been validated with the data from^16^. The drain current of the simulated heterojunction TMD TFET is in excellent match with the data obtained from^16^ as shown in (Fig. 5).

### Strain analysis

**Fig. 6:**
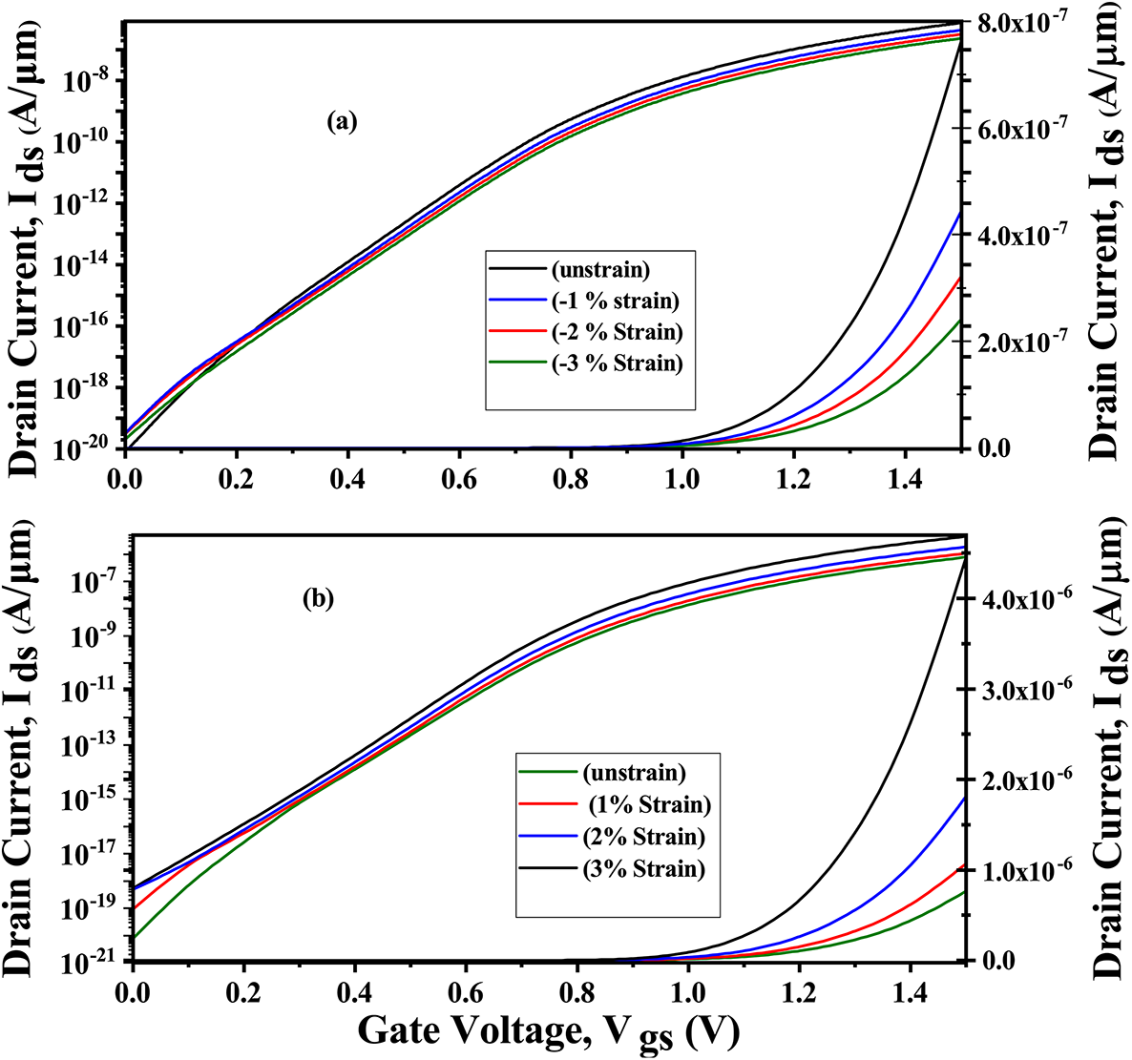
Impact of compressive and tensile strain on drain current for biomolecule having dielectric constant (k = 9).

At the interface, the heterostructure is formed by lattice mismatch due to the difference in lattice constants between the materials involved, resulting in strain. This section examines the effects of strain on the electrical properties of the proposed CP-TMJ-TFET based biosensor. First-principle density functional theory (DFT) calculations have been performed in Quantum Espresso using plane wave basis^22^. The first step was to relax the unit cells of both materials until the combined force was less than 0.001 Ry/a.u and the stress was less than 10^−7^ Ry/a0 (a0 being the Bohr radius). 50 Ry is chosen as the kinetic cut-off energy for the plane wave expansion. GGA proposed by Perdew-Burke-Ernzerhof (PBE) has been considered within Projected-Augmented Wave (PAW) pseudo-potential for exchange correlation function^23^. A 4×4×1 Monkhorst-Pack k-point grid samples the first Brillouin zone for unit cell optimizations and band-structure calculations. In lattice optimization, the energy convergence criterion is set at 10^−6^ eV, while the force convergence criterion is 0.001 eV/Å. The relaxed lattice constant a for MoS_2_ and WTe_2_ are a=3.19 and a=3.55 Å, respectively. Then, the WTe_2_-MoS_2_ heterostructure is created. The heterostructure’s relaxed lattice constant is a = 3.44 Å. The lattice constant of the WTe_2_-MoS_2_ heterostructure is greater than the standalone lattice constant of MoS_2_. Whereas, it is lesser than the standalone lattice constant of WTe_2_. The lattice constant at the WTe_2_-MoS_2_ heterostructure has been altered due to the lattice mismatch. To match the lattice constant at the interface, tensile strain up to (+1, +2, +3, +4, +5%) is applied to MoS_2_ and compressive strain up to (−1, −2, −3%) is applied to WTe_2_ along the transport direction of heterostructure, and, then, the calculated band-gap is fed to SILVACO TCAD for further simulations. Table 2 depicts the variation in band-gap of WTe_2_-MoS_2_ heterostructure with the variation in the nature of uniaxial strain. The strain can be calculated as,

1 $$S = \left( {\frac{{a - a_{o} }}{{a_{o} }}} \right) \; \times \;100\%$$

where, a and a_*o*_ are lattice constants of strained and unstrained structure. The I_*ds*_-V_*gs*_ characteristics for uniaxial tensile strain up to (−1, −2, −3%) and compressive strain upto (+1, +2, +3%) is shown in (Fig. 6a,b). The application of tensile strain improves the ON current over unstrained and compressively strained devices.

**Table 2:** Variation in the bangap with the application of applied uniaxial strain.

Uniaxial Strain (%)	Band-gap (eV)
−3	1.15
−2	1.25
−1	1.4
0	1.2
1	1.1
2	0.8
3	0.7

**Fig. 7:**
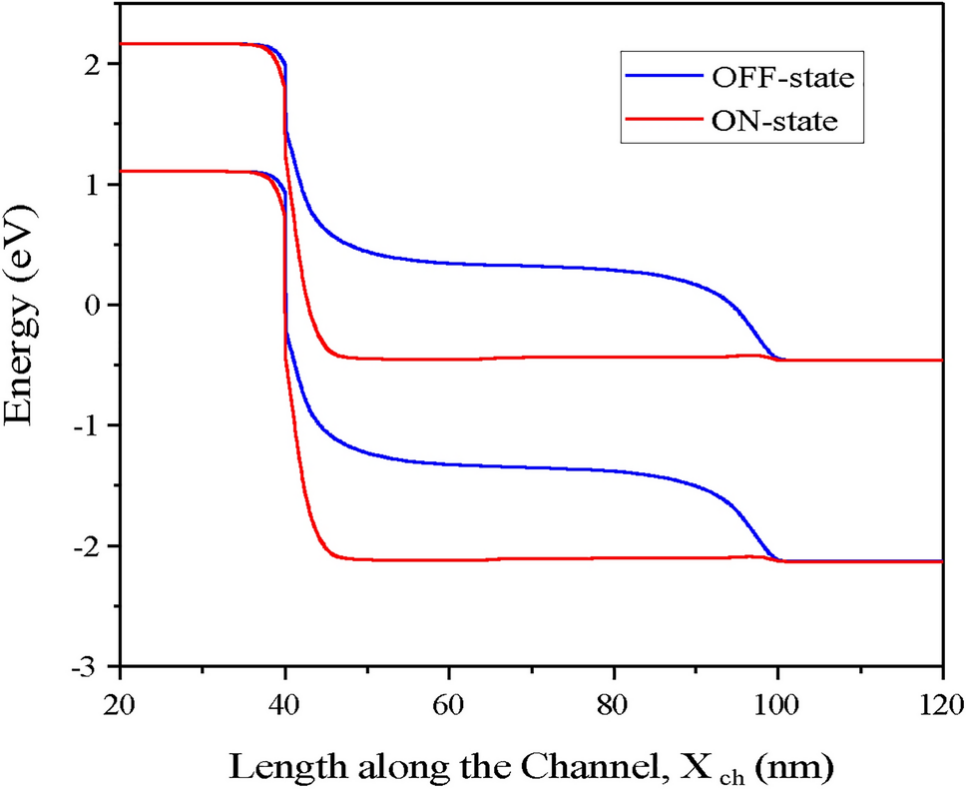
Energy band diagram of CP-TMD-HJ-TFET biosensor along the length of the channel for OFF-state and ON-state for neutral biomolecules having dielectric constant, (k = 9).

The energy band diagram of the CP-TMD-HJ-TFET-based biosensor in the ON-state for (V_*gs*_ = 1.5 V, V_*ds*_ = 0.5 V) and OFF-state for (V_*gs*_ = 0 V, V_*ds*_ = 0.5 V) is shown in (Fig. 7). Since the tunneling barrier length from source valence band to channel conduction band is wider under the OFF state, the electrons will need more energy to tunnel through the source-channel junction.

**Fig. 8:**
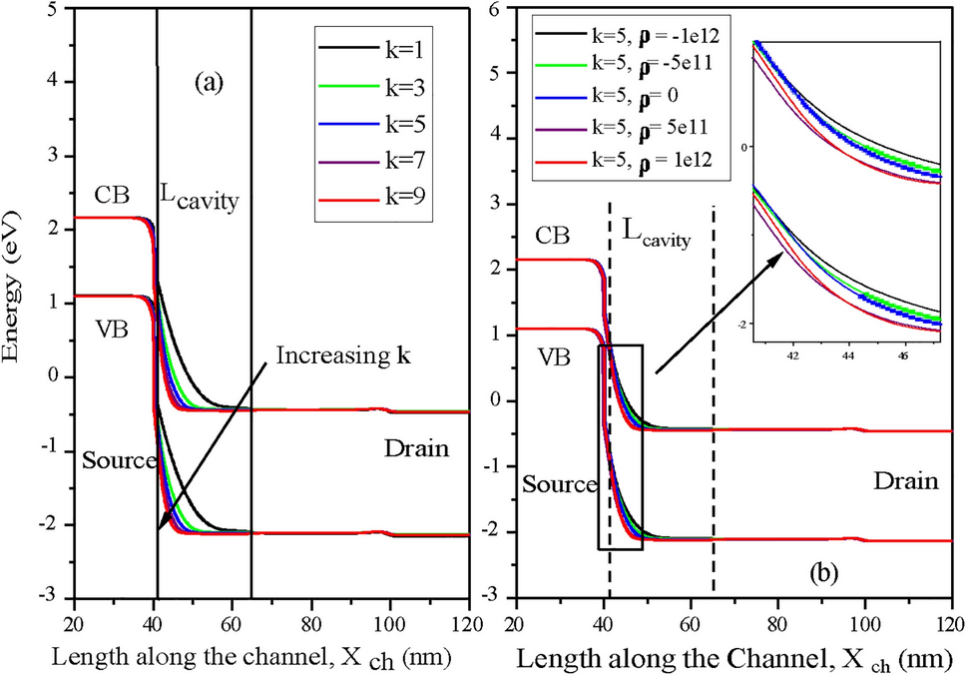
(**a**) Energy band diagram of CP-TMD-HJ-TFET biosensor along the length of the channel at V_*gs*_ = 1.5 V, V_*ds*_ = 0.5 V for biomolecules with dielectric constants, k = 3 to 9, *ρ* = 0. (**b**) Energy band diagram of CP-TMD-HJ-TFET biosensor along the channel-length at V_*gs*_ = 1.5 V, V_*ds*_ = 0.5 V for different charge densities (C/cm^2^) of biomolecules.

The energy band diagram along the channel-length for the variation in the dielectric constant (k=1 to 9) of neutral biomolecules is shown in (Fig. 8a). It depicts that the band bending under the cavity increases as the dielectric constant of biomolecules increases. This results in increase of the effective gate capacitance, and, thus coupling between gate and channel improves. It leads to reduction of the tunneling barrier length from the source valance band to channel conduction band. Figure 8b shows the energy band diagram along the length of the channel for the variation in charge density for biomolecules at a dielectric constant of (k=5). According to Gauss’s law, the negative/positive biomolecules in cavity will induce positive/negative charges at the surface, and it results in less/more band bending at the source-cavity interface due to p-type source.

**Fig. 9:**
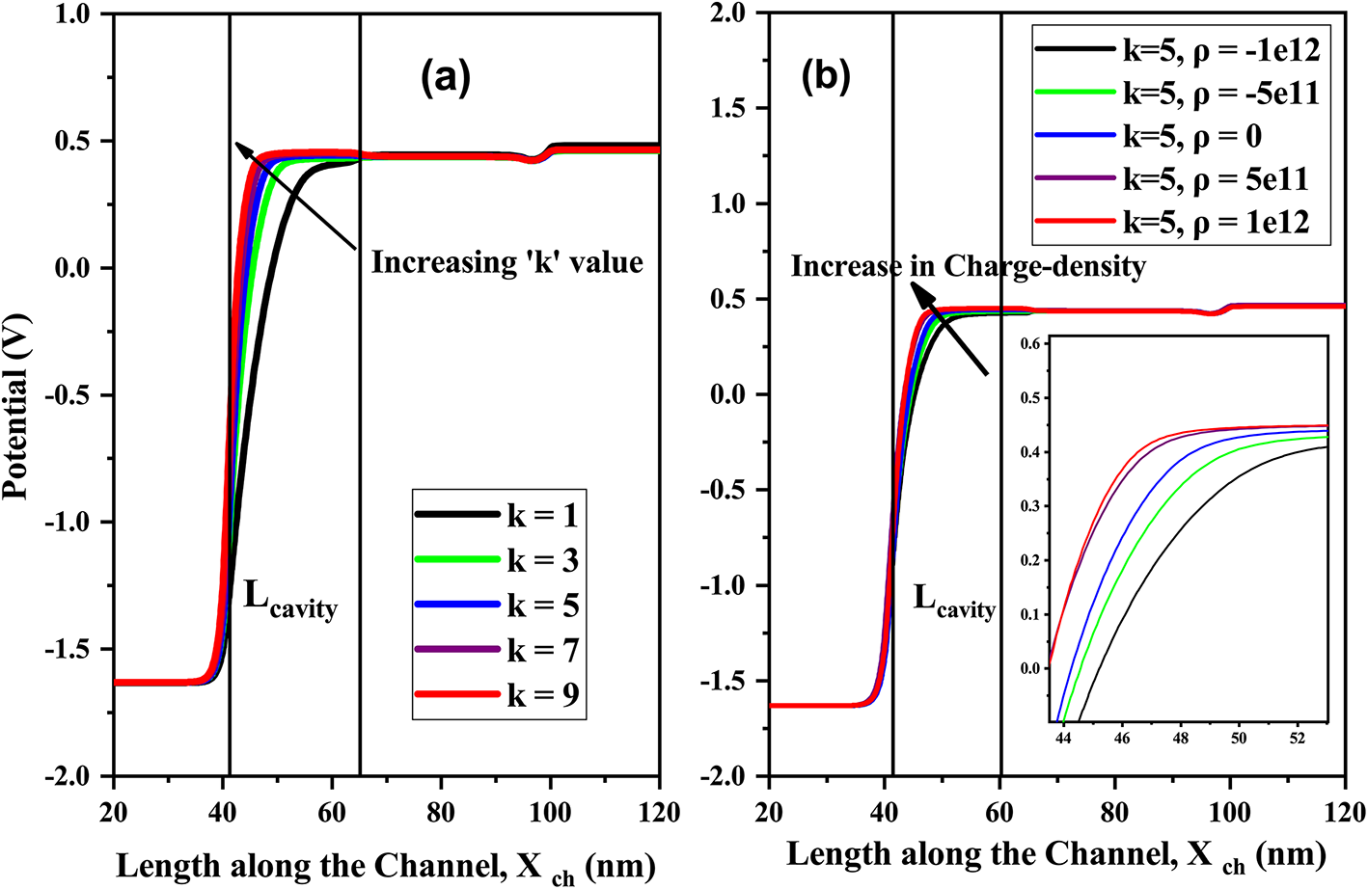
(**a**) Surface potential plot of CP-TMD-HJ-TFET biosensor along the channel-length at V_*gs*_ = 1.5 V, V_*ds*_ = 0.5 V for various dielectric constants (k) of biomolecules, (*ρ* = 0). (**b**) Surface potential plot of CP-TMD-HJ-TFET biosensor along the channel-length at V_*gs*_ = 1.5 V, V_*ds*_ = 0.5 V for different charge densities (C/cm^2^).

The surface potential of the channel for variation in the dielectric constants of the biomolecules is shown in (Fig. 9a). The surface potential beneath the cavity is lower when the cavity is empty (k = 1). As the dielectric constants (k>1) of biomolecules increases, the tunneling barrier length at the source-cavity junction is reduced, this phenomenon causes the surface potential to rise significantly^4^.

The surface potential over the length of the channel for various charge densities of biomolecules is depicted in (Fig. 9b). As the amount of negatively charged biomolecules increases in the cavity, the flat band voltage rises by a factor of (q*ρ*/C)^2^. As a result, the surface potential beneath the cavity region reduces. In the cavity, the flat band voltage reduces by the amount of (q*ρ*/C) with positive charge density. Thus, there is rise in surface potential for positively charged biomolecules. The relationship between effective gate capacitance, charge density of biomolecules and effective gate voltage is represented as^2^,

2 $$V_{GSef\;f} = V_{GS} - \left( {V_{FB} - \left( {{{q\rho } \mathord{\left/ {\vphantom {{q\rho } {C_{ef\;f} }}} \right. \kern-0pt} {C_{ef\;f} }}} \right)} \right)$$

**Fig. 10:**
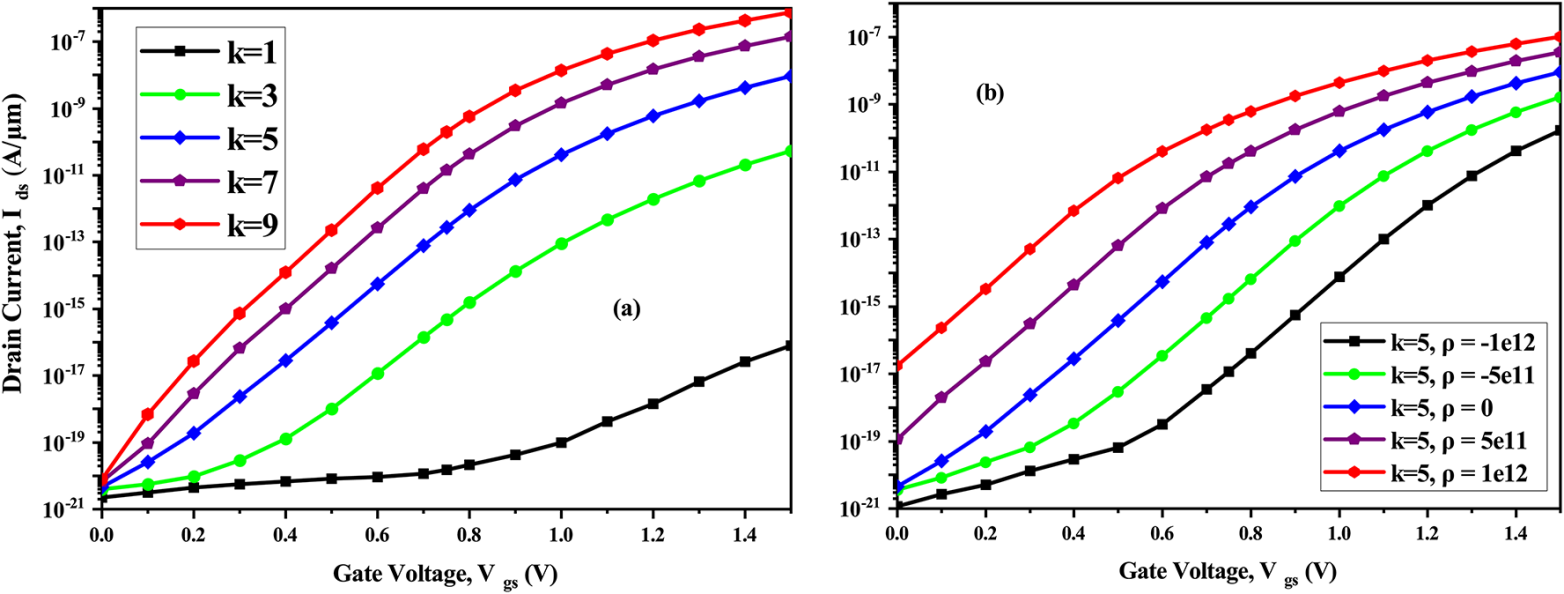
(**a**) I_*ds*_ vs V_*gs*_ of CP-TMD-HJ-TFET biosensor for various dielectric constant of biomolecules (*ρ* = 0) at V_*ds*_ = 0.5 V and (**b**) I_*ds*_ vs V_*gs*_ of CP-TMD-HJ-TFET biosensor for various charge densities (C/cm^2^) of biomolecules at V_*ds*_ = 0.5 V.

Figure 10a is the I_*ds*_-V_*gs*_ characteristics for different dielectric constants of biomolecules, (*ρ*=0). As dielectric constant increases from (k=1 to 9), there is an increase in the tunneling of electrons which leads to rise in the drain current. Drain current characteristics as a function of charge density of biomolecule is shown in (Fig. 10b). The energy band diagram in Fig. 8 shows that when the negative charge density increases, band-bending at the source-cavity interface decreases resulting in device conduction at higher V_*gs*_. From Fig. 10b, it can be concluded that for negatively charged biomolecules, device remains in the subthreshold region till ∼ V_*gs*_= 0.6 V, and, after this, the device conduction initiates and there is degradation in drain current. Whereas, band bending at the source-cavity interface increases for positively charged biomolecules, and the drain current rises.

**Fig. 11:**
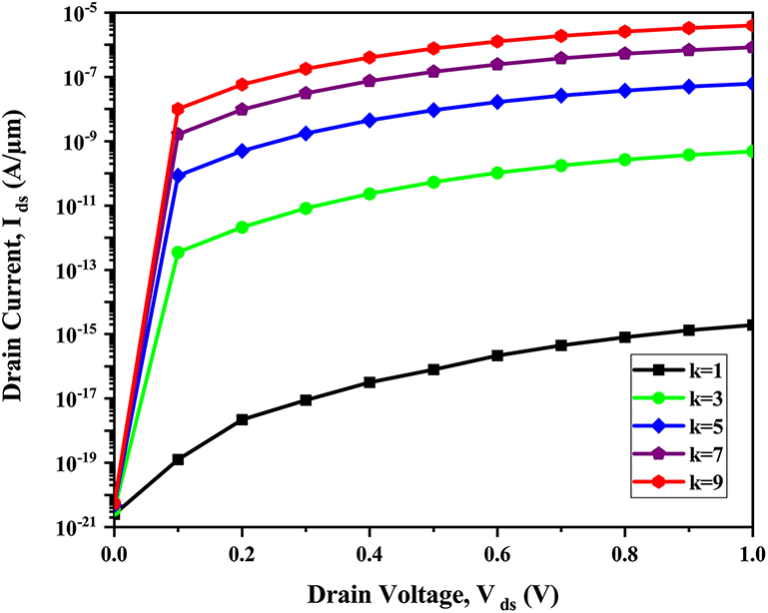
I_*ds*_ vs V_*ds*_ of CP-TMD-HJ-TFET biosensor for various dielectric constant values of biomolecules (*ρ* = 0) at V_*gs*_ = 1.5 V.

Figure 11 shows the I_*ds*_-V_*ds*_ plot for various dielectric constants of biomolecules with the variation in V_*ds*_.

Any biosensor’s sensitivity needs to be evaluated in order to identify the presence of specific biomolecules. The properties of the drain current alter as a result of the conjugation of biomolecules inside the cavity. The biosensor’s sensitivity can be measured using this variation in drain current. The drain current sensitivity expression used in this work is as follows^4,24,25^:

3 $$S_{Current} = \left( {\frac{{I_{ds}^{\;\;\;bio} - I_{ds}^{\;\;\;air} }}{{I_{ds}^{\;\;\;air} }}} \right)$$

**Fig. 12:**
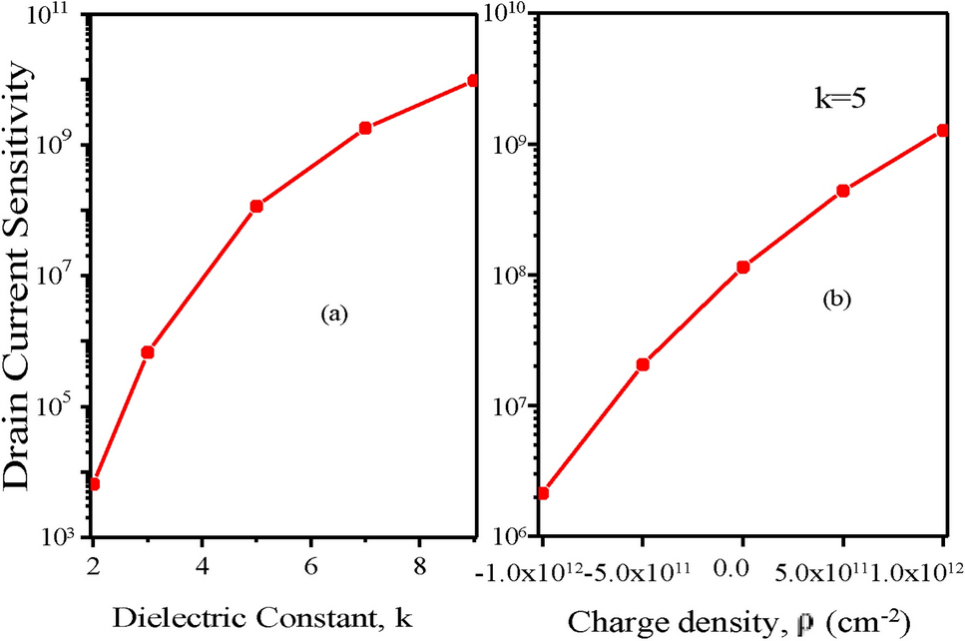
Drain Current Sensitivity of CP-TMD-HJ-TFET biosensor for variation in (**a**) the dielectric constants of the biomolecules (*ρ* = 0) and (**b**) for variation in charge density (C/cm^2^) of biomolecules.

**Fig. 13:**
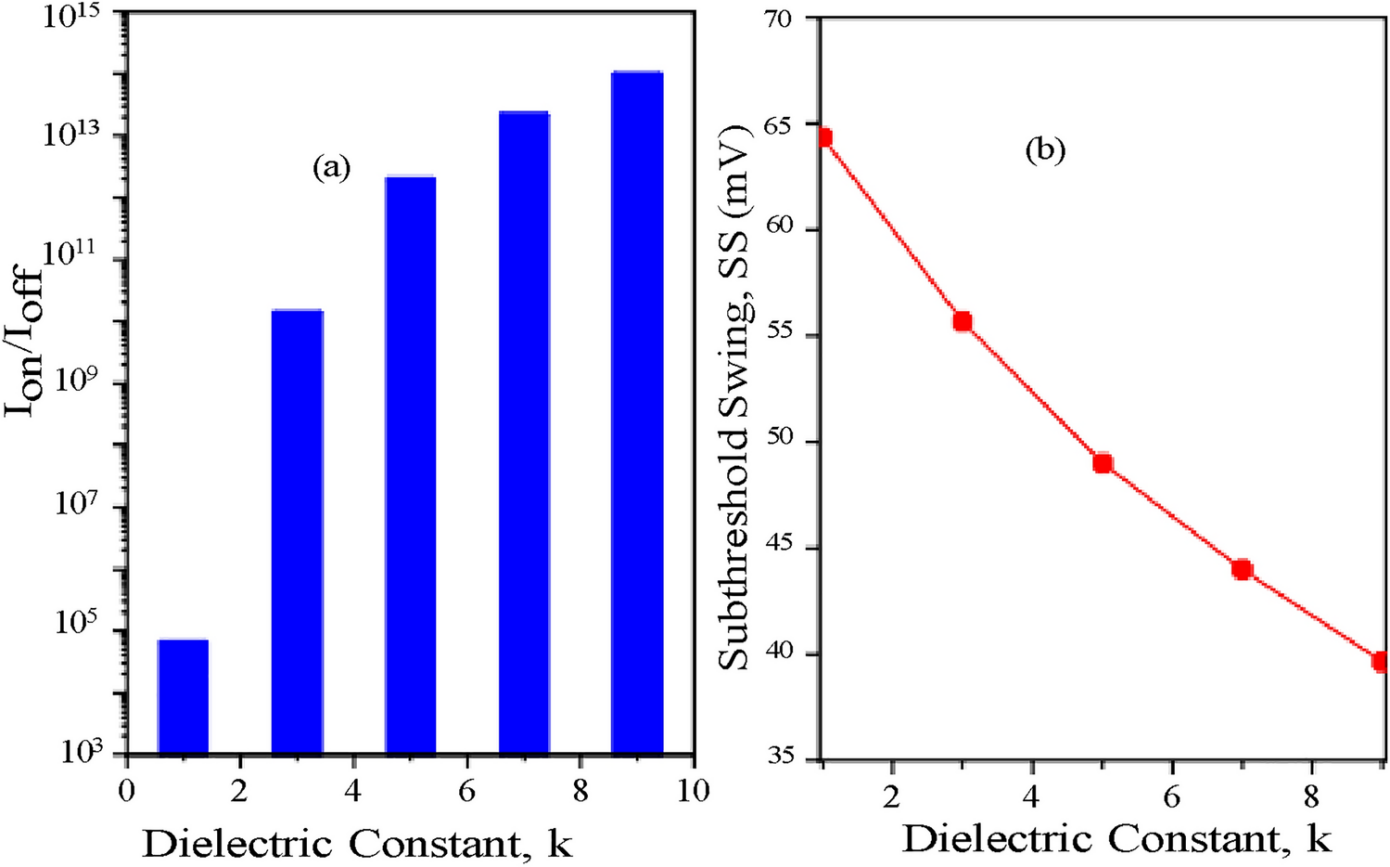
(**a**) I_*ON*_ to I_*OFF*_ ratio of CP-TMD-HJ-TFET biosensor for different dielectric constants of neutral biomolecules and (**b**) Subthreshold Swing of CP-TMD-HJ-TFET biosensor for variation in dielectric constants of neutral biomolecules.

When biomolecules are present inside the cavity, I_*ds*_^*bio*^ is drain current during ON state. I_*ds*_^*air*^ is ON state drain current when there are no biomolecules present inside the cavity. The relationship between the drain current sensitivity and the variation in dielectric constant (k) of biomolecules is shown in (Fig. 12a). There will be a simultaneous increase in drain current sensitivity as the drain current rises along with the biomolecules’ dielectric constant. The suggested CP-TMD-HJ-TFET biosensor for the dielectric constant (k=9) here, achieves a high drain current sensitivity of 10^10^. Figure 12b presents the relationship between the drain current sensitivity and the fluctuation in charged biomolecules density. In Fig. 12b, it is shown that negatively charged biomolecules result in a lower drain current sensitivity since their ON current is also lower. On the other hand for biomolecules with a positive charge, the sensitivity is more than the negatively charged biomolecules because the ON current is higher for the positively charged biomolecules. In Fig. 13a, the ratio of I_*ON*_/I_*OFF*_ is depicted. Increment in dielectric constant (k>1) for biomolecules reduces the tunneling barrier length sharply which leads to increment in the alignment between source valence band and channel conduction band. Thereby, drain current increases and I_*ON*_/I_*OFF*_ ratio rises profoundly when compared with I_*ON*_/I_*OFF*_ ratio at (k=1). For, CP-TMD-HJ-TFET biosensor, a high I_*ON*_/I_*OFF*_ ratio of 10^14^ is achieved for neutral biomolecules of dielectric constant (k=9). Subthreshold-Swing (SS) is a critical parameter for determining the effectiveness of a biosensor. The SS of a TFET-based biosensor is denoted as^18^,

4 $$SS = \frac{\Delta Vgs }{{\Delta \left( {log_{{{1}0}} I_{ds} } \right)}}mV/decade$$

Subthreshold Swing for variation in dielectric constants, (k=1 to 9) of neutral biomolecules is depicted in (Fig. 13b). It is depicted that with the increase in dielectric constants, subthreshold swing is reduced because the coupling between the gate and channel increases and tunneling barrier length decreases. Furthermore, when the biomolecules are immobilized in the cavity, the subthreshold swing falls below the MOSFET thermionic limit of 60 *mV* /decade. The proposed biosensor for biomolecules with a dielectric constant of (k=9) achieves a low subthreshold swing of 39 *mV* /decade.

**Fig. 14:**
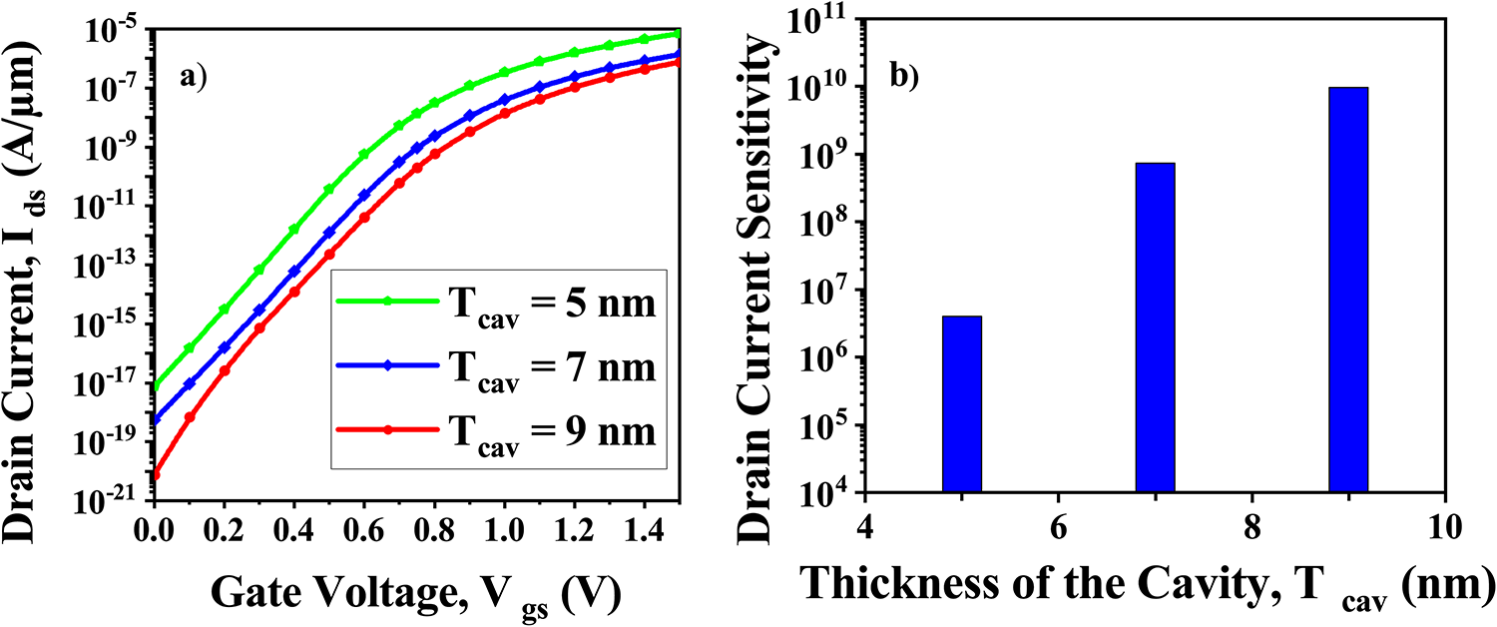
CP-TMD-HJ-TFET biosensor for the variation in cavity thickness at V_*ds*_ = 0.5 V for (k = 9) neutral biomolecule (**a**) I_*ds*_-V_*gs*_ and (**b**) Drain current sensitivity.

**Fig. 15:**
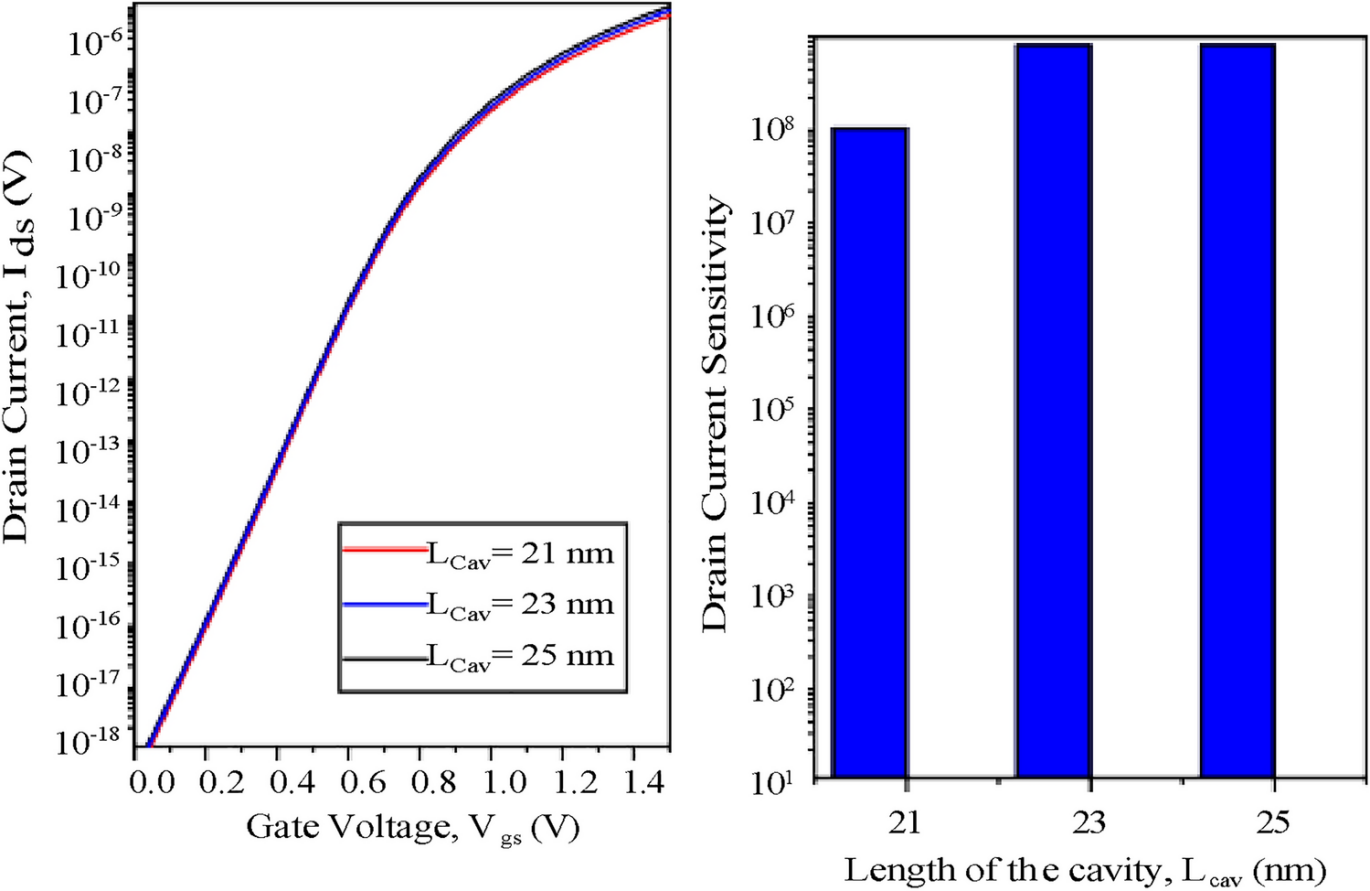
CP-TMD-HJ-TFET biosensor for the variation in cavity length at V_*ds*_ = 0.5 V for (k = 9) neutral biomolecule (**a**) I_*ds*_-V_*gs*_ and (**b**) Drain current sensitivity.

Because the operating mechanism of TFET-based biosensor is based on tunneling rather than diffusion, which is the phenomenon of traditional FET-based biosensors. Hence, variation in cavity length of the proposed biosensor does not have much of an effect on transfer characteristics and sensitivity^4^ as shown in (Fig. 14). According to I_*ds*_-V_*gs*_ characteristics for the change in cavity thickness, shown in Fig. 15a, the tunneling barrier width of the source-cavity junction increases as cavity thickness increases. It causes a rise in the gate to source voltage, V_*gs*_ required for device conduction, resulting in a fall in drain current. The drain current sensitivity is depicted in Fig. 15b along with the cavity’s thickness variation. As the cavity thickness increases, the drain current sensitivity rises.

### Impact of partial filling and percentage filling of biomolecules

**Fig. 16:**
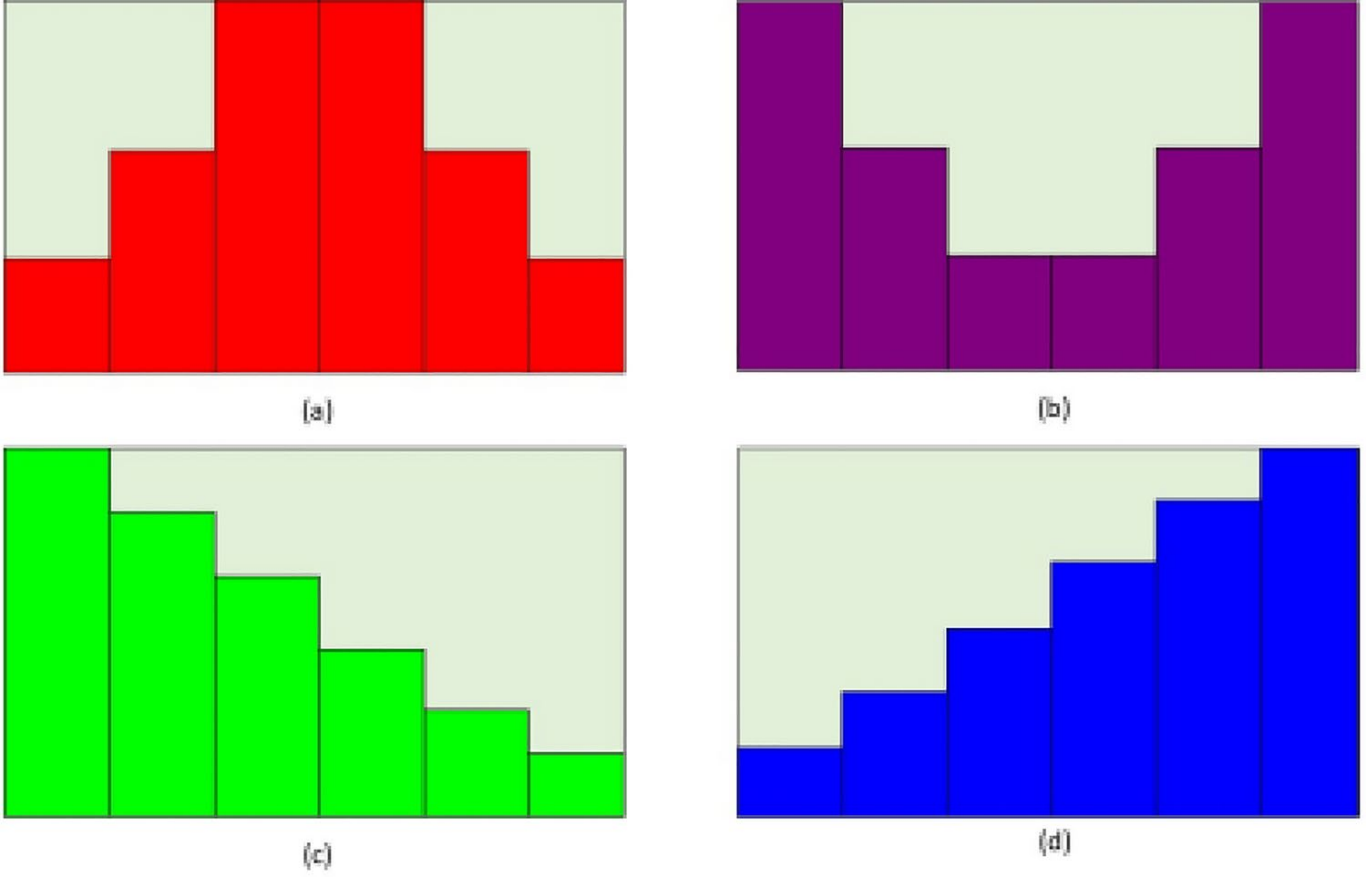
Profiles of the partially filled cavity: (**a**) Convex step profile, (**b**) Concave step profile, (**c**) Decreasing step profile and (**d**) Increasing step profile.

**Fig. 17:**
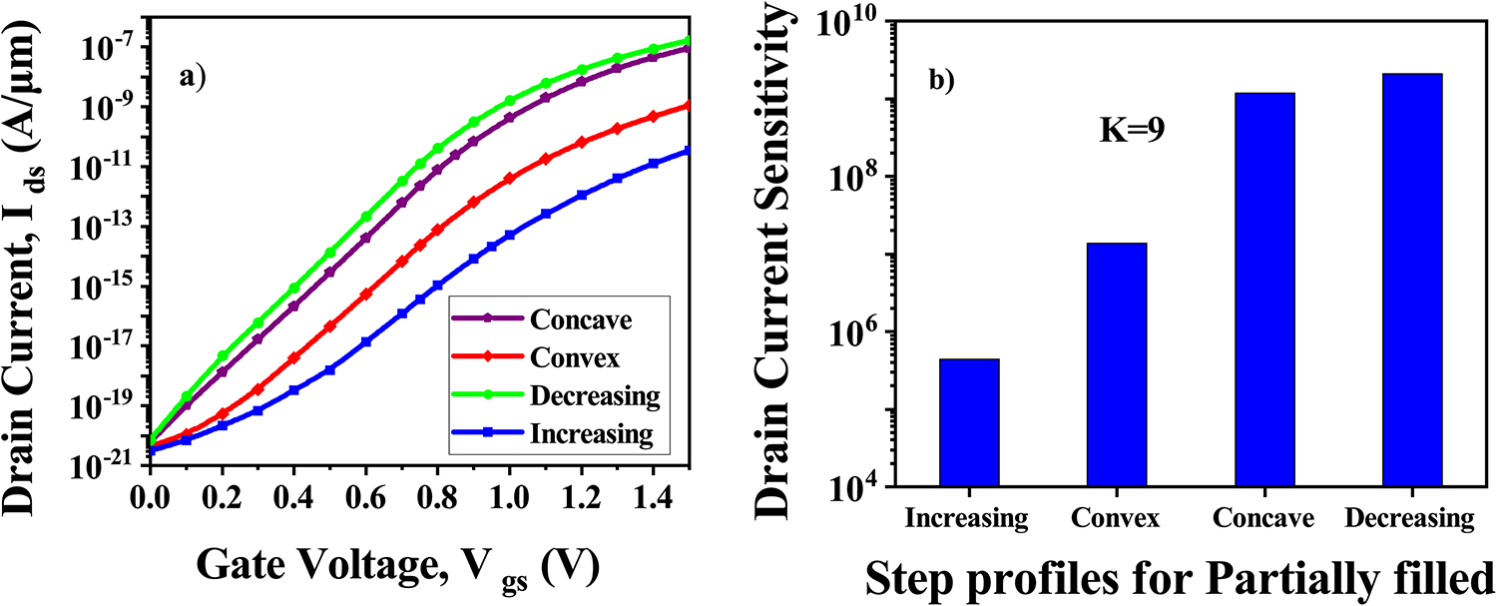
CP-TMD-HJ-TFET biosensor for different step profiles of the partially filled cavity at V_*ds*_ = 0.5 V for k = 9 neutral biomolecules (**a**) I_*ds*_ vs V_*gs*_ and (**b**) Drain current sensitivity.

In a realistic environment, it is not possible for cavities to be completely filled. In order to facilitate biosensing operations, biomolecules are immobilized and hybridized within the cavity. Hybridized existing biomolecules impede the entry of new biomolecules during biomolecule hybridization. This steric hindrance effect may cause biomolecules to hybridize in non- uniform ways along the length of the cavities, according to^15^. Convex step profiles Fig. 16a, concave step profiles Fig. 16b, decreasing step profiles Fig. 16c, and increasing step profiles Fig. 16d are four alternative partially filled cavity scenarios that are taken into consideration. Fig. 17a displays the I_*ds*_-V_*gs*_ characteristics for various step profiles of the partially filled cavity at V_*ds*_=0.5 V, while Fig. 17b displays the drain current sensitivity for various step profiles of the partially filled cavity. In the four scenarios mentioned above, the decreasing profile and concave step profile are more sensitive. For the decreasing profile and concave step profile, the drop in sensitivity is reduced when compared with the completely-filled cavity, but it is greater than the increasing profile and convex step profile. This is because the maximum biomolecule conjugation occurs close to the source/cavity tunnel junction in declining and concave profiles. As a result, the effective gate capacitance surrounding the junction is higher, which, in comparison, results in better sensitivity in partially filled scenarios. In general, the step profile with larger steps closer to the tunneling junction has a higher gate-channel coupling^15^.

**Fig. 18:**
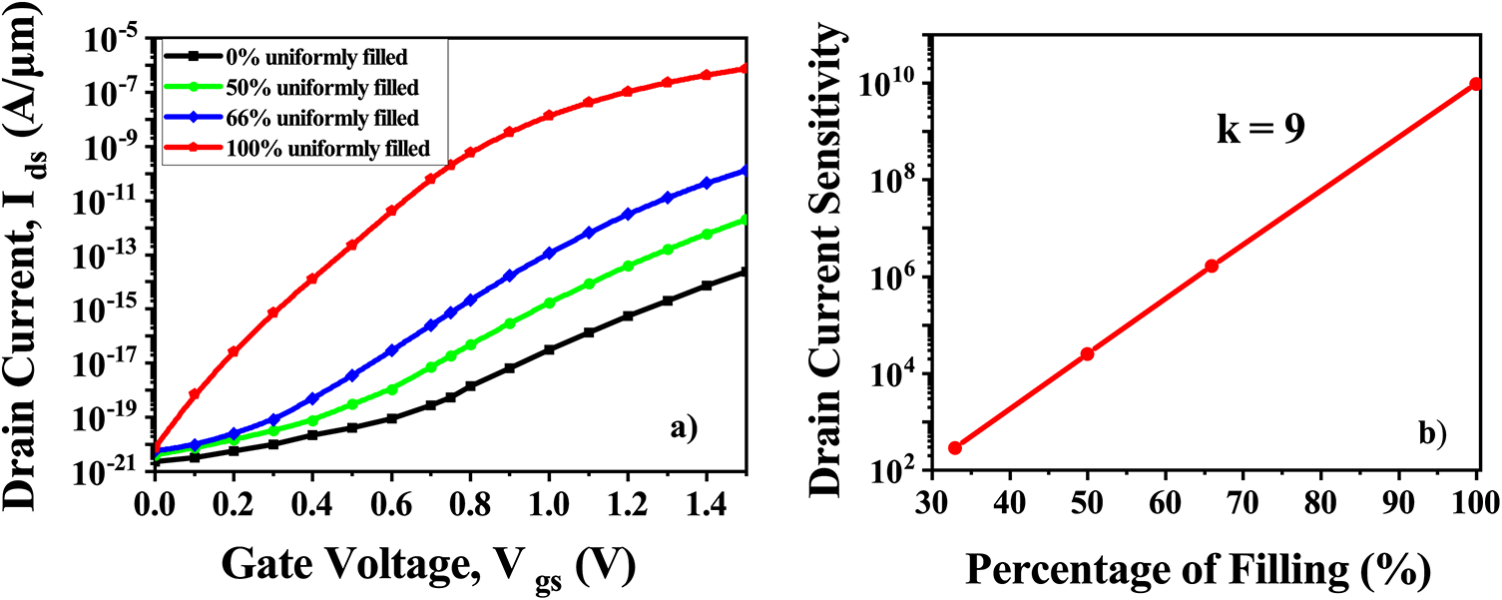
CP-TMD-HJ-TFET biosensor for percentage filling of the cavity at V_*ds*_ = 0.5 V and k = 9 (**a**) I_*ds*_ vs V_*gs*_ and (**b**) Drain current sensitivity.

**Fig. 19:**
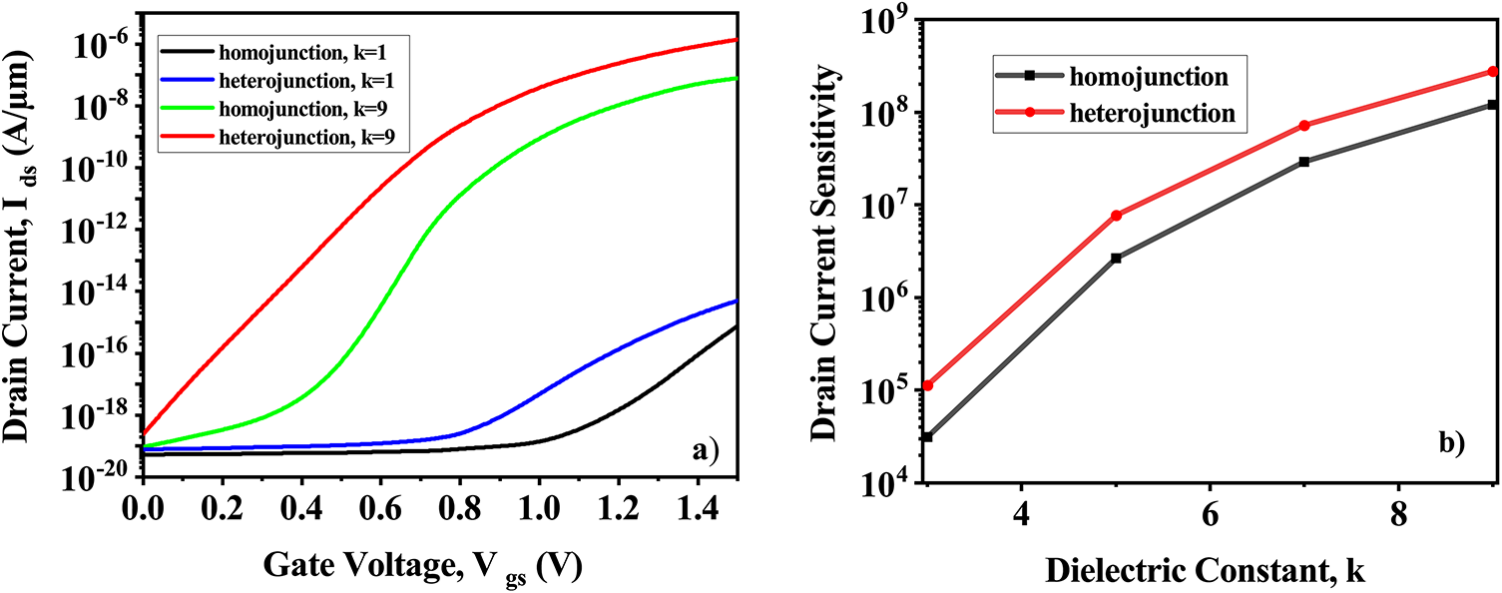
Comparison of homo and heterojunction CP-TFET based biosensor (**a**) Comparison of drain current of CP-TMD-HJ-TFET biosensor with CP-TMD-TFET biosensor and (**b**) Comparison of drain current sensitivity of CP-TMD-HJ-TFET biosensor with CP-TMD-TFET biosensor.

The suggested CP-TMD-HJ-TFET-based biosensor has also been carefully examined for the variation in the percentage of biomolecules present inside the cavity. The percentage filling of the cavity when, biomolecules with a dielectric constant of (k = 9) become immobilized at V_*ds*_ = 0.5 V, is depicted in (Fig. 18a). Along the length of the cavity, the filling is considered to be uniform. The amount of cavity filling has a big impact on the current. Figure 18b is the drain current sensitivity for the proportion of cavity filling. With respect to filling percentage, the sensitivity varies linearly. At a cavity thickness of 7 nm, Fig. 19 compares the Charge Plasma-based TMD homojunction TFET biosensor with a Charge Plasma-based TMD heterojunction TFET biosensor. Charge Plasma-based TMD homojunction Tunnel FET biosensor is formed by keeping MoS_2_ in all the regions (Source, Channel, Drain). Because of the lower band-gap material i.e. WTe_2_ in source in the case of heterojunction, the tunneling barrier length is smaller, resulting in more tunneling and a higher drain current, as shown in (Fig. 19a). The sensitivity of both homojunction and heterojunction Charge Plasma-based TMD TFET biosensors is shown in (Fig. 19b). The Charge Plasma-based TMD heterojunction TFET biosensor has a higher drain current sensitivity than the Charge Plasma-based TMD homojunction TFET biosensor. As presented in Table 3, the proposed CP-TMD-HJ-TFET-based biosensor has been benchmarked against the most advanced biosensors from recent literatures. It is clear that the suggested biosensor exhibits greater sensitivity of 10^10^ when compared with the contemporary state-of-the-art biosensors.

**Table 3:** Benchmarking with contemporary works of literatures.

Biosensor	Sensitivity Parame- ters	Bias and k value	Our work
Ref^24^	4.78 × 10^7^	V_*ds*_ = 1 V, V_*gs*_ = 1.5 V, k = 10	3.67 × 10^9^
Ref^33^	2.7 × 10^5^	V_*ds*_ = 1 V, V_*gs*_ = 1.5 V, k = 10	3.67 × 10^9^
Ref^34^	5.55 × 10^2^	V_*ds*_ = 0.5 V, V_*gs*_ = 1.5 V k = 10	1.74 × 10^10^
Ref^4^	8.9 × 10^5^	V_*ds*_ = 0.5 V, V_*gs*_ = 1.5 V k = 10	1.74 × 10^10^
Ref^25^	1 × 10^5^	V_*ds*_ = 0.5 V, V_*gs*_ = 1.5 V k = 11	2.85 × 10^10^
Ref^15^	2 × 10^7^	V_*ds*_ = 0.5 V, V_*gs*_ = 1.5 V, k = 10	1.74 × 10^10^
Ref^35^	1.13 × 10^10^	V_*ds*_ = 0.5 V, V_*gs*_ = 1.5 V, k = 12	4.78 × 10^10^
Ref^36^	1.1 × 10^6^	–	4.78 × 10^10^
Ref^37^	2.94 × 10^9^	V_*ds*_ = 0.5 V, V_*gs*_ = 1.5 V, k = 12	4.78 × 10^10^

## Modelling and simulation methods

Simulation of TMD-material based TFETs requires a full quantum simulation of the device. In this work the charge plasma homo-junction TMD-TFET-based biosensor is simulated using atomistic simulator NanoTCAD ViDES^26^ within the non-equilibrium Green’s function (NEGF) formalism. The surface potential of the CP-TMD-TFET-based biosensor has been validated against the simulation data obtained from SILVACO TCAD. Since atomistic simulator has high complexities, the charge plasma heterojunction TFET based biosensor is modeled using SILVACO TCAD^27^ as it has a lower computational complexity and also offers high accuracy, so it is better suited for a thorough analysis of the proposed device properties. In^28–32^ SILVACO TCAD simulator was utilized to simulate the properties of TMD MOSFETs and TFETs using drift-diffusion approach. They are modeled using the material parameters available in the literature and considering the 3-D equivalent of their 2-D density of states. When defining 2-D materials in SILVACO TCAD, it is necessary to define the material permittivity, band-gap, density of states, electron and hole mobilities, electron and hole effective masses, and affinity. The relevant material parameters of WTe_2_ and MoS_2_ considered in the simulation has been extracted from first principal simulation using Quantum espresso and has been validated with^28^ is shown in (Table 4).

**Table 4:** Relevant parameters of monolayer MoS_2_ and WTe_2_ considered in TCAD simulations.

Parameters	MoS_2_	WTe_2_
Band-gap *E*_*g*_ (in eV)	1.62	1.06
Electron affinity *χ* (in eV)	4.28	3.75
Dielectric Constant (*κ*)	4.8	7.18
Effective mass of elec-tron (*m*_*e*_)	0.47	0.33
Effective mass of holes (*m*_*h*_)	0.57	0.41
Mobility (cm^2^/V.s)	320	125

TFETs exhibit nearly ideal switching behavior in the absence of parasitic effects. We simulated the proposed Charge Plasma Hetero TFET-based biosensor under ideal conditions and introduced trap-assisted tunneling (TAT) models to account for realistic scenarios in charge plasma home junction TFET biosensor simulations and verified using NanoTCAD ViDES. Our model incorporated non-local band-to-band tunneling to calculate tunneling currents, considering spatial variations within energy bands. Recombination phenomena were included using the SRH model, and mobility was described using the CVT model, accounting for transverse field, doping, and temperature effects. Fermi–Dirac statistics were implemented using the FERMI model. Additionally, quantum tunneling meshing techniques were applied to accurately model tunneling regions, with the source/channel tunneling junction meshed at a higher density for precise electrical property simulation.

## Conclusion

In this work, Charge Plasma TMD Heterojunction TFET-based biosensor has been designed and different sensitivity parameters have been estimated for biosensing applications. It has been determined that the CP-TMD-HJ-TFET biosensor has a very high sensitivity of 10^10^ (for k = 9), a superior I_*ON*_/I_*OFF*_ ratio of 10^14^ (for k = 9), and a subthreshold swing of 39 mV /decade, which is lesser than the MOSFET-based biosensors’ limit of 60 mV /decade. The cavity thickness has been optimized, which improved the I_*ON*_ and sensitivity of the device. Partial filling of the cavity has also been considered for practical scenarios. It has been discovered that the percentage and position of biomolecules within the cavity have a significant impact on the efficacy of the proposed biosensor. When compared to the charge plasma-based TMD homojunction TFET biosensor, the proposed Charge Plasma TMD heterojunction TFET-based biosensor has shown superior sensitivity. In conclusion, the proposed.

CP-TMD-HJ-TFET demonstrates a notable improvement in sensitivity by ∼ 4 decades of magnitude compared to current state-of-the-art biosensors, making it highly effective for label-free detection of biomolecules, even at low concentrations.

## Data Availability

Data can be available upon reasonable request to the corresponding author.
